# Using Patient-Reported Outcome Measures for Improved Decision-Making in Patients with Gastrointestinal Cancer – the Last Clinical Frontier in Surgical Oncology?

**DOI:** 10.3389/fonc.2013.00157

**Published:** 2013-06-14

**Authors:** Kjetil Søreide, Annbjørg H. Søreide

**Affiliations:** ^1^Department of Gastrointestinal Surgery, Stavanger University Hospital, Stavanger, Norway; ^2^Department of Clinical Medicine, University of Bergen, Bergen, Norway; ^3^Stokka Geriatric Care Facility, Stavanger Municipality, Stavanger, Norway

**Keywords:** patient-reported outcomes, quality of life, cancer, surgery, research

## Abstract

The genomic era has introduced concepts of “personalized medicine” and “targeted therapy” in the field of oncology. Medicine has become increasingly complex with a plethora of potential dilemmas in diagnosis, treatment, and management. The focus on classical outcomes for clinical decision-making is now increasingly being replaced by patient-reported outcome measures (PROMs). PROMs should increasingly now be in the center of patient-centered decision-making, based on valid, reliable, and clinically useful measures delivered directly by the patient to the caregiver. Surgeons’ ability to interpret and apply PROMs and quality of life results must improve by education and further research, and has an unreleased potential to contribute to a better understanding of the patients’ well-being. A number of caveats must be addressed before this can be brought to fruition; standardization for valid items; appropriate use of instruments; correct timing of the application; missing data handling, compliance, and respondent drop-outs are but a few issues to be addressed. Based on the apparent lack of use in both research and clinical work, it should call for an educational effort to address this among surgeons caring for patients with cancer.

## Introduction

The genomic era has swept the medical community since the beginning of the twenty-first century, leading to an advent of “personalized medicine” and “targeted therapy” in particular within the field of oncology. The opportunities are increasing together with the survival rates, as are the cost of care. The outcome measures of the past, usually measured as hard endpoints in mortality and morbidity (“M&M”), are no longer as valid as they used to be. That is – while “M&M” should still be at their lowest possible rates – traditional “M&M” are no longer viewed as a complete measure of “success” of any given treatment alone, such as evaluation of outcomes for surgery for cancer. Rather, the patient-reported outcomes should have an increased role in decision-making, but must be based on valid, reliable, and clinically useful measures (Lipscomb et al., 2007).

Indeed, medicine has become increasingly complex and with complexity a plethora of choices and potential dilemmas in diagnosis, treatment, and management of disease – for both doctor as well as the patient. With increasing knowledge and available options, uncertainty may prevail over confidence and communication and interaction in the patient-doctor relationship may suffer (Srivastava, 2011). Critical appraisal of the potential benefits and harms of the given options, within the context of the patient’s characteristics, conditions, and preferences has thus become increasingly important. The “new-kid-on-the-block” in research methodology is the approach to “patient-centered outcomes” or “patient-reported outcomes” in the managed care for patients – used as determinant of improvement of care and areas for directed attention of care. As a form of practice, it seeks to focus medical attention on the individual patient’s needs and concerns, rather than the doctor’s (Bardes, 2012). The growing demands for quality and safety in health care have refocused attention on patient outcomes(Lipscomb et al., 2007; Sloan et al., 2007; Trotti et al., 2007; Wagner et al., 2007; Blazeby, 2010; Williamson et al., 2012a; Macefield et al., 2013). This perspectives article will briefly discuss the rationale; the definitions and perceived concepts; the need for standards; issues related to surgical oncology; and, eventually, some future directions for patient-centered outcomes measures in health research.

## The Rationale: Patient-Centered Outcomes as Basis for Improved Care

Several arguments are available for the need to embark on PCO research (Gabriel and Normand, 2012). For one, the growing and aging population present with increasingly complex medical health problems. The primary disease under treatment may not be the only disease needed to take into consideration, but also other comorbid conditions that may be influenced by the choice of treatment. As such, the need for integrating knowledge about PCO in the discussion with patients becomes instrumental. Second, the available treatment options have increased both in types and forms, with different effects and potential side-effects for drugs or interventions aimed at the same disease. Thus, weighing the benefits and risks for each individual should take into account the preferred knowledge about expected patient-oriented outcomes. Third, the requirements to cost-effectiveness, resource use, and priority in different health systems require an explicit knowledge on how exactly the health systems requirement influence care on the patient level. Finally, the individualized approach to modern medicine with targeted therapies based on a person’s genetic and epigenetic profile comes along with the need to know how certain other patient’s characteristics may influence the response and outcome of specific therapies on a individual level rather than a group level. Each of these points requires the development of a rigorous methodology in order to arrive at valid and reliable answers. While this methodology is under development still, the need for an evidence-based overview of available methodology, endorsement of accepted methods, and guidelines for recommended methods has become evident. In response to this, a Patient-Centered Outcomes Research Institute (PCORI) has been created in the USA to support research that can produce answers generated with the use of rigorous, valid, patient-centered methods (Washington and Lipstein, 2011; Gabriel and Normand, 2012). The PCOR Institute has adopted the following mission statement to guide their work: “… *help people make informed healthcare decisions, and improves healthcare delivery and outcomes, by producing and promoting high-integrity, evidence-based information that comes from research guided by patients, caregivers, and the broader healthcare community*” (Washington and Lipstein, 2011). Further, developing and improving the science and methods of comparative clinical effectiveness research and producing high methodological standards for research are among the current tasks the institute is working on. A recently released draft of recommendations for selected standards for the conduct of research leading to evidence-based, patient-centered health interventions has been reported (www.pcori.org). The report describes the rationale behind creating standards for patient centeredness, for prioritizing topics for research, for choosing a study design, and for designing, conducting, and reporting patient-centered outcomes research (Gabriel and Normand, 2012). Clearly, a new “movement” has come of age in terms of research agenda and priority. Patient-centered outcomes research will have a focus that ranges from the individual patient and help patients make informed decisions, but will also focus on dissemination of knowledge, alleviate disparities and focus on improvement of health systems in a broader sense. Truly it makes sense to include patient-centered information for improved patient-centered care. But, the tools for doing this are not yet widely standardized nor implemented across ongoing clinical investigations.

## Definitions and Concepts of Patient-Centered Outcomes Research

**Figure 1:**
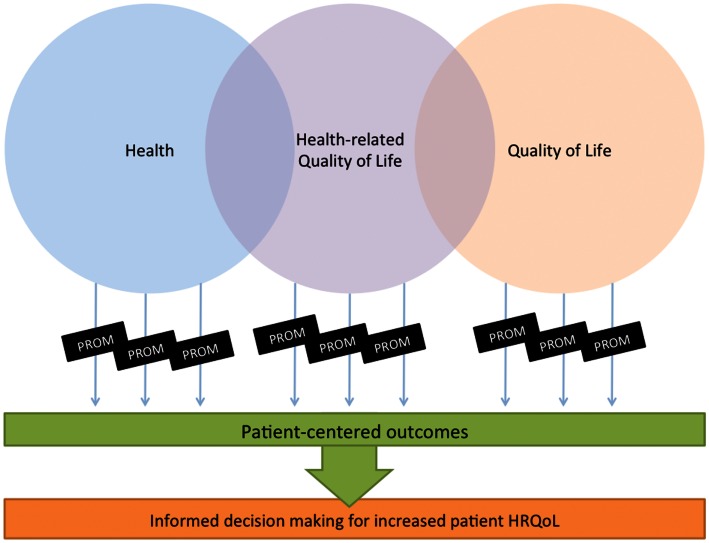
**Relation between measures of interest**.

A much discussed and debated definition; “health” is not the mere absence of disease, but moreover “health” refers to the state of human physical, social, and emotional well-being. Further, “quality of life” (QoL) may be viewed as any aspect of health, life satisfaction, or happiness. Then, “health-related QoL” is a multidimensional assessment of health. In this figure (Figure 1) of overlapping definitions, a “patient-reported outcome measure” (PROM) may refer to a self-report (by the patient) of one or more aspects of health. As is given, a PROM may be derived from any of the health assessment fields, but may be much more narrow focused, such as typically a focus on pain as a PROM, or a cluster of symptoms to define a specific domain, or the “symptom burden” (Cleeland, 2007; Jeffery et al., 2009).

**Figure 2:**
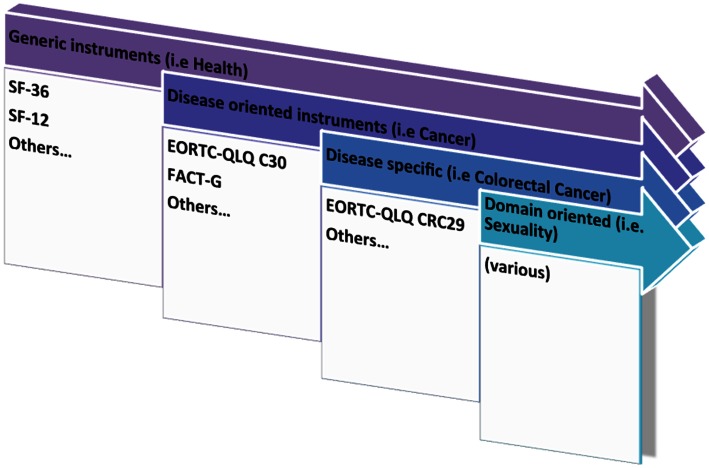
**Approaches to PROMs research – from generic to specific**.

Consequently, three main approaches to the measurement of PROMs in patients with cancer may be considered (Hadi et al., 2010), as depicted in Figure 2. First, the generic approach to health status measurement allows for the comparison across health conditions; secondly, the cancer-generic modules which are more focused on dimensions relevant to people with cancer (Luckett, 2011) and which may allow comparison of scores across groups with cancer (of different or similar origin); and, lastly, any add-on modules which focus more on specific domains relative to the type of cancer, i.e., colorectal cancer (Whistance et al., 2009) or, liver metastasis from colorectal cancer (Blazeby et al., 2009). Also, one may consider specific domain-related instruments (i.e., fatigue; sexuality) within or beyond the disease-specific domains.

Independent of this, it is important that the patient outcome of investigation comes directly from the patient (Fiscella et al., 2011), without the filtration or interpretation of information or graded response by an investigator or anyone else. PROMs can be investigated in either generic terms (i.e., overall/global health evaluation) or with regard to more disease-specific aspects (i.e., cancer-specific measures).

## Standards for PROM Research

While there are several benefits and ways of investigating PROMs, there are equally many pitfalls and failures to this type of research, of which a few shall be further addressed. Among the benefits are the ability to use PROMs for assessment of nature of disease or treatment effects not amenable to other scores; the ability to obtain the patients own perception, rather than the health professionals’ judgments (which may often deviate from the patient); PROMs may give reliable prognostic info as demonstrated in several studies (Montazeri, 2009); and inform economic analyses (Blazeby, 2010). More specifically, PROMs may inform decision-making (Hughes et al., 2012) to the benefits of surgery in settings where surgery may compete with non-surgical alternatives, or in settings were surgery may only pose a marginal benefit or even the potential for harm (Avery et al., 2008; Blazeby, 2010).

Among the troubling areas in this field is the different use and availability of tools (Whistance et al., 2009; Cleeland and Sloan, 2010; Colwell et al., 2010; Macefield et al., 2013) – in some areas there may be a lack of standardized and validated instruments, while in other areas there may be instruments designed for the same or similar purpose (Luckett, 2011), which may compete and even clutter the interpretation of results obtained across studies.

Further, the availability and validity of instruments has been a problem, with no standards for translational processes from one language to another. This has now been rectified to some extent by proposals of standards among others by organizations, such as the EORTC (Koller et al., 2007). Also, increasingly validation studies are performed across cancer types (Blazeby et al., 2009). Yet, still, when trying to compare studies there is considerable heterogeneity according to the PROMs used, the frequency, content, and presentation of feedback, and the training offered to medical teams for using PROMs (Luckett et al., 2009). Further, trials varied in their unit of randomization, outcome measures, control of contamination, monitoring of PROM use, and length of follow-up (Luckett et al., 2009). Obviously, there is a need for future interventions to ensure that PROM data are used to optimum effect and for trials to control for contamination and monitor use of PROMs to link this with outcomes.

## PROMs in Surgical Cancer Care and Research

One should recognize that QoL investigation and the use of PROMs has different applications to patients with advanced cancers compared to those undergoing curative treatment. Historically, QoL issues may have had a stronger focus in advanced cancer care and palliative medicine (Cella, 1995; Bottomley et al., 2005; Groenvold et al., 2006; Byrne et al., 2007; Abernethy et al., 2010; Cella et al., 2011; Amdal et al., 2013), but is now gaining importance in several fields for which curative surgery may be the central point in the care bundle for patients with cancer (Tan et al., 2012; Williamson et al., 2012a,b; Macefield et al., 2013; Pusic et al., 2013; Winters et al., 2013). Patient satisfaction (as a PROM) with hospital care is indeed independent of morbidity, treatment type, and QOL outcomes (Avery et al., 2006) – emphasizing the fact that PROMs may capture feedback information to providers not easily obtained through other metrics. In contrast to other fields, one should recognize that surgery in itself may have immediate and long-lasting effects on PROMs and correspondingly HRQoL measures, both in generic terms and also for disease-specific inventories.

In clinical terms, surgeons or the associated caregiver can use the results of PROM instruments to track patients’ functional status and QoL changes through treatment, or in the follow-up of a given treatment (Morris, 2006). Obtaining formalized and “objective” results (although based on the patients “subjective” report) might help surgeons and physicians to better communicate with patients during their treatment, especially with regard to patients’ expectations. Knowing that numerous clinical variables of uncertain value are regularly followed after cancer surgery such as for colorectal cancer (Kørner et al., 2005; Søreide et al., 2012), it is troublesome to know that validated PROM/QoL instruments have been put to use only rarely in the clinical setting.

A recent review (Macefield et al., 2013), found six systematic reviews reporting on the evaluation of PROM in colon or rectal cancer, two on esophageal cancer, and one on upper-GI cancers in general (esophagogastric and pancreatobiliary). The authors found that the systematic reviews found several limitations in PROM reporting, heterogeneity in the use of PROMs (which hampered comparison between studies) and overall very few studies (four of eight RCTs on colorectal cancer; two of two trials on esophageal cancer) were of satisfactory quality to draw somewhat robust conclusions.

## Future Directions

Patient-reported outcome measures research has proved to be highly wanted for current modern research on clinical outcomes. It is thus important that this be performed in a standardized and proper manner. Standardizations and suggestions to improve this is currently underway. The Task Force of The International Society of QoL Research is working on a CONSORT extension for reporting PROMs in randomized controlled trials (Calvert et al., 2011) which may inform and structure the standards for using health-related QoL in RCTS as either a primary or secondary outcome (Brundage et al., 2012). As may be a problem across several types of research, problems with proper sample size calculations have been tangible in PROMs research as well. A recent review have quantified the outcomes which should form the basis for more reliable sample size calculation in the future (Cocks et al., 2011), but still a major problem may be the agreement on type of PROM, the measured (or reported) effect and the size in the difference in measurements for making clinically useful interpretations. Obviously, education on the user level is mandatory, as is ensuring the agreement on proper timing for PROM assessment (Ediebah et al., 2013). Compliance remains an issue in this kind of research as in many other fields (Land et al., 2007), and better understanding of drop-out rates and tools to potentially improve compliance and reduce drop-outs is needed. Last but not least, the proper analyses of obtained data, including pre-study preparation of methodology and dealing with missing data. Also, recommended methods (Macefield et al., 2013) of communicating patient-reported outcome and clinical data to patients (e.g., by means of visually displayed outcomes or distributions) should be followed so as to gain as widely clinical use of the obtained info for each and every patient as possible.

Clearly, PROMs should be a prioritized area of current clinical research not only in the palliative setting but more so for patients receiving surgical care of curative intent for cancer. Surgeons’ ability to interpret and apply PROMs and QoL results will surely improve with practice, and has an unreleased potential to contribute to a better understanding of the patients’ well-being.

## Conflict of Interest Statement

The authors declare that the research was conducted in the absence of any commercial or financial relationships that could be construed as a potential conflict of interest.
